# Association of Cardiovascular Health with Epicardial Adipose Tissue and Intima Media Thickness: The Kardiovize Study

**DOI:** 10.3390/jcm7050113

**Published:** 2018-05-13

**Authors:** Jana Hruskova, Andrea Maugeri, Helena Podroužková, Tatiana Štípalová, Juraj Jakubík, Martina Barchitta, Jose R. Medina-Inojosa, Martin Homolka, Antonella Agodi, Sarka Kunzova, Ondrej Sochor, Francisco Lopez-Jimenez, Manlio Vinciguerra

**Affiliations:** 1 International Clinical Research Center, St’Anne University Hospital, Brno 60200, Czech Republic; jana.hruskova@fnusa.cz (J.H.); andreamaugeri88@gmail.com or andrea.maugeri@fnusa.cz (A.M.); juraj.jakubik@fnusa.cz (J.J.); martin.homolka@fnusa.cz (M.H.); sarka.kunzova@fnusa.cz (S.K.); ondrej.sochor@fnusa.cz (O.S.); 2 Department of Medical and Surgical Sciences and Advanced Technologies “GF Ingrassia”, University of Catania, 95123 Catania, Italy; martina.barchitta@unict.it (M.B.); agodia@unict.it (A.A.); 3 Department of Internal Medicine – Cardioangiology, Faculty of Medicine, Masaryk University, Brno 65691, Czech Republic; helena.podrouzkova@fnusa.cz (H.P.); 177418@mail.muni.cz (T.S.); 4 Division of Preventive Cardiology, Department of Cardiovascular Medicine, Mayo Clinic, Rochester, MN 55905, USA; medinaInojosa.Jose@mayo.edu (J.R.M.-I.); lopez@mayo.edu (F.L.-J.); 5 Division of Medicine, University College London (UCL), London, NW3 2PF, UK

**Keywords:** cardiovascular risk, epicardial adipose tissue, intima media thickness

## Abstract

**Background:** Intima-media thickness (IMT) has been proposed as a measurement of subclinical atherosclerosis and has been associated with cardiovascular disease (CVD). Epicardial adipose tissue (EAT) is a fat depot between the pericardium and myocardium and has been associated with coronary atherosclerosis. The relationship between IMT and EAT thickness has not been reported before. We investigated the relationship between EAT thickness, IMT, CVD risk factors, and ideal cardiovascular health (CVH) metrics using subjects from the Kardiovize Brno 2030 cohort study, a random urban sample population in Central Europe. **Methods:** We studied 102 individuals (65 males) aged 25–64 years (median = 37 years) with no current or past CVD history. We measured IMT using a vascular ultrasound and EAT thickness using transthoracic echocardiography, and collected data on anthropometric factors, CVD risk factors, and CVH score. Correlation tests and multiple linear regression models were applied. **Results:** In the age- and gender-adjusted model, we demonstrated that, among CVD risk factors, only BMI was significantly and positively associated with EAT thickness (β = 0.182, SE = 0.082, *p* = 0.030), while no significant associations with IMT were evident. Although both EAT thickness and IMT were negatively correlated with CVH score (*r* = −0.45, *p* < 0.001, and *r* = −0.38, *p* < 0.001, respectively), we demonstrated that overall CVH score (β = −0.262; SE = 0.077; *p* = 0.001), as well as BMI (β = −1.305; SE = 0.194; *p* < 0.001) and blood pressure CVH metrics (β = −0.607; SE = 0.206; *p* = 0.004) were significantly associated with EAT thickness but not with IMT. **Conclusions:** Our study is important as it demonstrated for the first time that CVH is associated with EAT thickness. Interestingly, this relationship seems to be dependent on BMI and blood pressure rather than on the other CVH metrics. However, outcome-driven studies are required to confirm these findings.

## 1. Introduction

In 2010, the American Heart Association (AHAM, Dallas, TX, USA) defined the cardiovascular health (CVH) score, which is based on the levels of seven metrics (i.e., body mass index (BMI), healthy diet, physical activity, smoking status, blood pressure, blood glucose, and total cholesterol) [1]. This tool allows the identification of individuals with poor CVH (i.e., at least one of seven metrics at poor level) at higher risk of CVD 9. While obesity, or BMI > 30, is associated with significant cardiovascular morbidity and mortality and is acknowledged as a major public health concern linked to poor cardiovascular health (CVH) [2,3], the distribution pattern of excess adiposity, including ectopic fat deposition, may be a more reliable indicator of cardiovascular disease (CVD) risk [4]. Ectopic fat deposition occurs around blood vessels and internal organs and can have either systemic or local effects [5]. Epicardial adipose tissue (EAT) is visceral intra-pericardial fat, contiguous, and shares a microcirculatory supply with the myocardial surface. EAT is highly bioactive, secreting a range of pro- and anti- inflammatory adipokines [6]. EAT develops from embryonic brown adipose tissue and it exerts mechanic, metabolic, and thermogenic functions [7]. In pathological conditions, excess EAT has been associated with an increased risk of heart failure, insulin resistance, metabolic syndrome, atrial fibrillation, coronary atherosclerosis, and fatty liver disease [6], as well as with cognitive impairment in the elderly [8]. Carotid artery intima-media thickness (IMT) is a measure of early atherosclerosis and may help in the risk prediction of CVD beyond traditional risk factors measured at the baseline [9]. Thus, it is believed that an investigation of EAT and IMT might independently reflect pre-symptomatic stages of vascular disease, which could benefit the most from interventions. While the association between poor CVH and IMT has been demonstrated in the Multi-Ethnic Study of Atherosclerosis, conducted on 5961 subjects, which demonstrated an inverse association between CVH scores and IMT in all ethnic groups taken into account [10], there is a lack of evidence for EAT. Moreover, a comprehensive analysis of the relationship between CVH, IMT, and EAT thickness has yet to be performed, and it is not clear which of the CVH score components, besides obesity, is responsible for or associated with the increased values of these echocardiographic parameters in the population.

Such an analysis is much needed, as CVD is now the leading cause of death globally [11], and the identification of objective risk factors and easily measurable imaging outcomes could have immediate relevance for the public health strategy. Among developed countries, CVD has the highest incidence in Eastern and Central Europe [12]. To better understand the prevalence of cardiovascular risk factors in Central Europe we have conducted the Kardiovize Brno 2030 study, a prospective cohort recruited to investigate the complex relationships of CVD risk factors and outcomes with a range of biological, psychosocial, environmental, and behavioral factors in an urban population—Brno, the second largest city of the Czech Republic [13]. The first aim of the present study was to assess the association of age, sex, anthropometric measures, and CVD risk factors with IMT and EAT, among a subset of subjects in the Kardiovize Brno cohort study. Furthermore, we aimed to demonstrate the association of overall CVH score, as well as each of the seven CVH metrics, with the echocardiographic imaging parameters IMT and EAT thickness.

## 2. Methods

### 2.1. Study Design and Participants

The Kardiovize Brno cohort comprises a randomly-selected 1% sample (*n* = 2160) of the city of Brno residents aged 25–64 years (mean age of 47 ± 11.3 years), which is assessed for traditional and novel CVD risk factors [13]. Health assessments and CVD risk data collection were performed by trained nurses and physicians at St Anne’s University Hospital in Brno, using the web-based research electronic data capture (REDCap) database [14]. A face-to-face questionnaire-based health interview was carried out for collecting information on: (i) demographic and socioeconomic status (age, gender and education); (ii) cardiovascular risk behaviors (smoking status, nutrition, alcohol consumption, and physical activity); and (iii) personal and family medical history (diseases, medications, and hospitalizations) [13].

The present cross-sectional study was conducted on a sub-sample of the Kardiovize Brno cohort. One hundred and two subjects with no previous or current history of CVDs, undergoing deep echocardiographic measurement and imaging, were eligible for the current analysis. In this sub-sample, we also measured both traditional and novel CVD risk factors.

### 2.2. Anthropometric Assessment

All anthropometric measurements were performed by trained researchers according to standardized techniques: height and weight were measured using a stadiometer (SECA 799; SECA, GmbH and Co. KG, Hamburg, Germany); waist, hip, and neck circumferences were measured manually with a tape; BMI (kg/m^2^) was calculated by dividing the person’s weight in kilograms by their height in meters squared. According to the World Health Organization protocol, waist-to-hip ratio (WHR) was calculated as the waist measurement divided by the hip measurement; central obesity was defined as WHR > 0.90 for men and WHR > 0.85 for women. Body fat mass was assessed using a direct segmental multi-frequency bioelectrical impedance analysis (InBody 370; BIOSPACE Co., Ltd., Seoul, Korea).

### 2.3. Physical Examination and Laboratory Analyses

The standard examination and analysis study protocol for Kardiovize Brno 2030 has been published previously [13] and was followed here. Blood pressure (BP) was measured using a mercury sphygmomanometer (Baumanometer, W.A. Baum, Co., Inc., Copiague, NY, USA) in three positions. Laboratory analyses were performed on 12-h fasting full blood samples using a Modular SWA P800 analyzer (Roche, Basel, Switzerland), with total cholesterol, triglycerides, glucose, and creatinine assayed by the enzymatic colorimetric method (Roche Diagnostics GmbH, Mannheim, Germany) and HDL-cholesterol by the homogeneous method for direct measuring without precipitation (Sekisui Medical, Hachimantai, Japan). The level of LDL-cholesterol was calculated according to the Friedewald equation when triglyceride levels were lower than 4.5 mmol/L; if higher, LDL-cholesterol was assayed by the homogeneous method for direct measuring (Sekisui Medical, Hachimantai, Japan). Urine albumin was assayed by immunoturbidimetry (Roche Diagnostics GmbH, Mannheim, Germany) in a morning spot urine sample, and the urinary albumin/creatinine ratio was calculated. The Ankle brachial index (ABI) is calculated as the ratio of the highest registered measurements of ankle and brachial blood pressures. Ankle and brachial pressures were measured with patients lying in the supine position. Doppler measurement of right brachial artery systolic pressures was performed 2 mm under the cuff, with the same measuring schema at ankle systolic pressure. Doppler-controlled artery flow and Doppler signals were acquired with the Dopplex SD2 device (HUNTLEIGH Healthcare Ltd., Cardiff, UK) using a VP5HS 5MHz transducer.

### 2.4. Ideal Cardiovascular Health Score

CVH score, as defined by the American Heart Association (AHA, Dallas, TX, USA) [1], is a sum measure of seven metrics: BMI, healthy diet, physical activity level, smoking status, blood pressure, blood glucose, and total cholesterol. Each metric is given a point score of 0, 1, or 2, depending on whether their value falls into the poor, intermediate, or ideal CVH-associated range, respectively; thus, the overall CVH score ranges from 0 to 14. Ideal CVH status requires all seven health metrics to be at ideal levels (score 14); intermediate CVH is defined as having at least one health metric at intermediate level, but no poor health metrics; and poor CVH as having at least one of seven health metrics scored as poor [15].

### 2.5. Echocardiographic Measurement of Epicardial Fat Thickness

Echocardiographic measurements were acquired with a GE- Vingmed Vivid E9 apparatus (GE Vingmed Ultrasound AS, Horten, Norway) using a 1,5–4,6 MHz sector transducer. Patients were placed in the lateral decubitus position and standard images were obtained from two-dimensional echocardiography, as recommended by the American Society of Echocardiography. All analyses were performed by EchoPAC PC software ver. 113. EAT thickness was measured with two-dimensional echocardiography, using the modified method based on previous studies [16]. The parasternal long-axis was modified (PLAX mod) to focus the right ventricle, and the parasternal short- axis (PSAX) was obtained at the level of the papillary muscle or the mitral valve. EAT was identified as the echo-free or hypoechogenic space between the outer wall of the myocardium and the visceral layer of the pericardium [17], and it was then measured as the maximum diameter of the hypoechogenic space between the myocardium of the right ventricle and the visceral pericardium. The area at the level of the aortic root was excluded from the measurement of EAT. EAT thickness was measured during the end-systole in three cardiac cycles. PAT was identified and defined as the maximum diameter of the hypoechogenic space between the visceral pericardium and mediastinum [18].

### 2.6. Measurement of Carotid Intima-Media Thickness

The Intima-Media Thickness (IMT) is the measure of the thickness of the carotid artery wall, achieved using B-mode ultrasound, and is a standard part of the assessment of atherosclerotic changes as recommended by The American Society of Echocardiography [19]. Ultrasound measurements were acquired with the ESAOTE MyLabClassC ultrasound (ESAOTE S.p.A, Genova, Italy) using the LA523 4-13MHz linear transducer. Patients were lying in the supine position with the neck rotated away from the examining physician. Both left and right Common Carotid Arteries (CCA) were measured, 1 cm proximal to their bifurcation. Evaluation of the IMT was performed by semi-automated ESAOTE MyLabClassC software using patented methods of analyzing RF data from the B-mode images.

### 2.7. Statistical Analyses

Statistical analyses were conducted using SPSS software (version 22.0, SPSS, Chicago, IL, USA). The Kolmogorov-Smirnov test was performed to assess the normal distribution of variables. Descriptive statistics were used to characterize the population, using frequency or mean and standard error of the mean (SE). Continuous variables underlying normal distribution were compared using analysis of covariance (ANCOVA), otherwise using nonparametric methods, adjusting for the effect of age. Categorical variables were compared using a Chi-square test. Correlation analysis was performed using Pearson or Spearman tests. The strength of correlation was interpreted based on the absolute value of the correlation coefficient as exhibiting a weak (*r* < 0.3), moderate (0.3≤ *r* < 0.7), or strong (*r* ≥ 0.7) correlation. Multiple linear regression models were used to evaluate the independent relationship of cardiovascular risk factors found to be correlated in bivariate analysis (predictors) with EAT thickness and IMT (dependent variables). Similarly, we assessed the relationship of overall CVH or seven CVH metrics (predictors) with EAT thickness and IMT (dependent variables). The models were unadjusted and adjusted for age (continuous) and gender (men vs. women). A *p* value < 0.05 was considered statistically significant in all analyses. 

## 3. Results

### 3.1. Study Population

Compared to the whole cohort, the sub-sample of the present analysis consisted of mostly male (63.7% vs. 45.2%; *p* < 0.001) and young (40.65 years vs. 47.30 years; *p* < 0.001) subjects, while no differences existed in terms of BMI, smoking status, and prevalence of hypertension, hyperlipidaemia, and diabetes. Specifically, the mean age was 38.51 years for men and 44.41 years for women (*p* = 0.004). The percentage of current smokers was 28.4%. The prevalence of hyperlipidaemia, hypertension, and diabetes mellitus was 74.7%, 36.3%, and 7.8%, respectively. No difference in the prevalence of hypertension, hyperlipidaemia, and diabetes was reported between men and women.

### 3.2. Differences in Anthropometric Characteristics and Cardiovascular Parameters by Gender

The age-adjusted anthropometric measures and cardiovascular parameters according to gender are presented in Supplemental Table S1. While BMI was comparable between sex (mean = 27.42 kg/m^2^, SE = 0.60 for men vs. mean = 26.25 kg/m^2^, SE = 0.80 for women), WHR was higher in men than in women (mean = 0.92, SE = 0.01 vs. mean = 0.79, SE = 0.01; *p* < 0.001), as well as body surface area (BSA; mean = 2.61 m^2^, SE = 0.03 vs. mean = 1.90 m^2^, SE = 0.04; *p* < 0.001) and the prevalence of central obesity (58.9% vs. 30.8%; *p* = 0.018). Fasting glucose mean values of 5.03 (SE = 0.07) mmol/L and 4.77 (SE = 0.09) mmol/L were found in male and female participants, respectively (*p* = 0.030). Compared to women, men also exhibited higher levels of triglycerides and creatinine (mean = 1.33 nmol/L, SE = 0.08 vs. mean = 0.99 nmol/L, SE = 0.11; *p* = 0.012 and mean = 13.37 nmol/L, SE = 0.74 vs. mean = 10.23 nmol/L, SE = 0.99; *p* = 0.015, respectively), and lower levels of HDL cholesterol (mean = 1.31 mmol/L, SE = 0.05 vs. mean = 1.72 mmol/L, SE = 0.07; *p* < 0.001). Statistically significant differences in IMT (mean = 559.98 mm, SE = 11.38 vs. mean = 519.39, SE = 15.97; *p* = 0.046), but not in ABI, between men and women were evident.

### 3.3. Ideal Cardiovascular Health and Score

We first examined whether the sub-sample included in the present analysis was representative of the whole Kardiovize Brno 2030 cohort, in terms of CVH. Accordingly, compared to the whole cohort, the sub-sample did not show differences in CVH score and metrics. 

**Figure 1:**
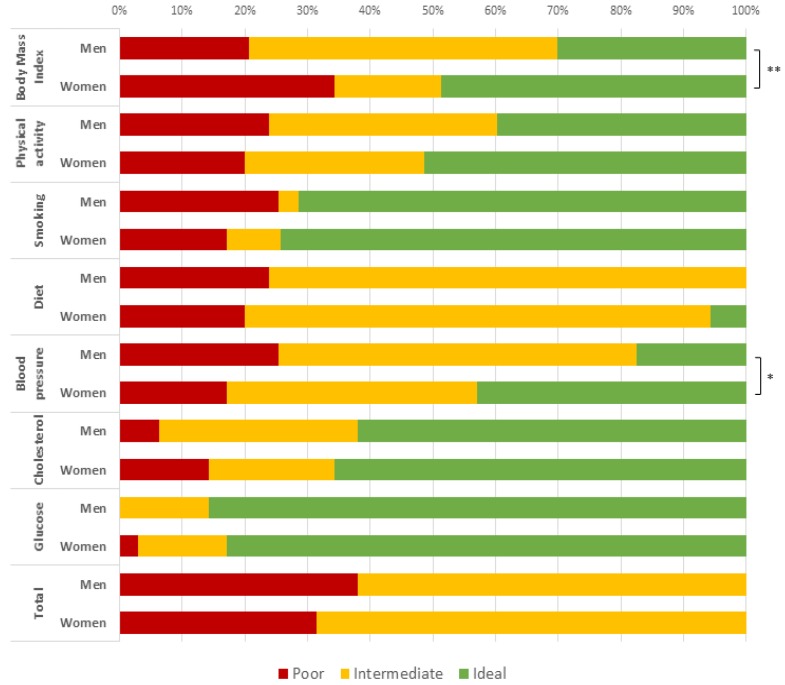
Distribution of ideal Cardiovascular Health (CVH) metrics stratified by sex, in the sample population (men = 65, women = 37).

Mean CVH score was 9.04 (SE = 0.24) and none of the participants scored “ideal” (all 7 CVH metrics at ideal levels) according to AHA definitions [1]. The prevalence of intermediate CVH (at least 1 intermediate metric and no poor metrics) was 35.7%. In contrast, 64.3% of participants had “poor” CVH (at least 1 poor health metric). Figure 1 shows the score distributions for each of the seven CVH metrics according to gender. Significant differences between men and women were reported in the distribution of BMI (*p* = 0.007) and blood pressure components (*p* = 0.024).

### 3.4. Associations of Anthropometric Measures and Cardiovascular Parameters with Epicardial Adipose Tissue (EAT) Thickness

**Figure 2:**
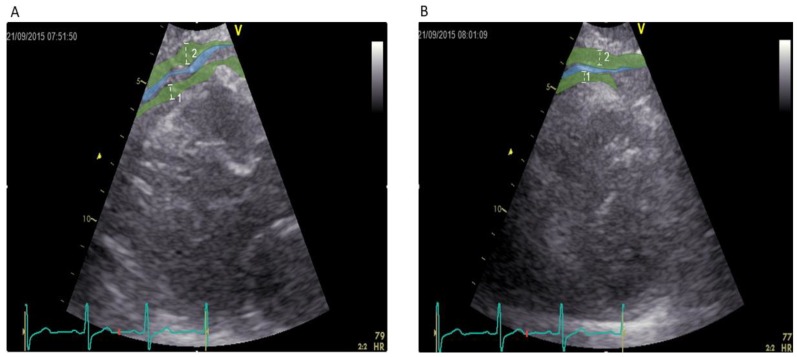
(**A**) Identification of tissues in parasternal long axis projection modified to focus on right ventricle (PLAX mod). Epicardial adipose tissue (EAT) thickness was identified as the maximum diameter (white line 1) of the hypoechogenic space between the myocardium of the right ventricle and the visceral pericardium (blue area). The area at the level of the aortic root is excluded from the measurement of EAT due to fact that EAT is physiologically present in this area. Paracardial adipose tissue (PAT) thickness was identified as the maximum diameter (white line 2) of the hypoechogenic space between the visceral pericardium (blue area) and the mediastinum. Both in the middle line along the midline of the ultrasound beam at end-systole; (**B**) Identification of tissues in parasternal short axis projection papillary muscle level (PSAX). Level of the PSAX was selected individually for every patient and is dependent on the identification point in the PLAX mod. EAT thickness was identified as the maximum diameter (white line 1) of the hypoechogenic space between the myocardium of the right ventricle and the visceral pericardium (blue area). The area at the level of the aortic root is excluded from the measurement of EAT due to fact that EAT is physiologically present in this area. PAT thickness was identified as the maximum diameter (white line 2) of the hypoechogenic space between the visceral pericardium (blue area) and the mediastinum. Both in the middle line along the midline of the ultrasound beam at end-systole.

**Figure 3:**
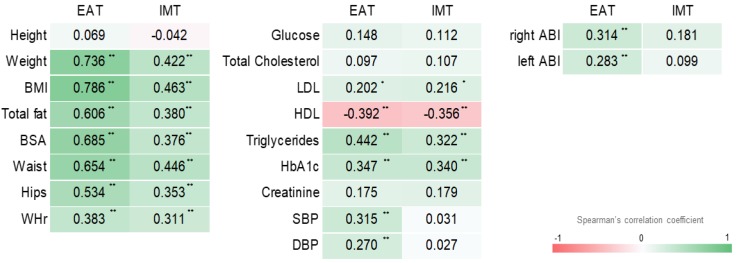
Heatmap representation of Spearman’s correlation coefficients of EAT thickness and IMT with anthropometric features (height; weight; body mass index, BMI; body surface area, BSA; total fat; waist; hip; waist-to-hip ratio, WHR), and cardiovascular parameters (glucose; total cholesterol; LDL; HDL; triglycerides; glycated hemoglobin, HbA1c; creatinine; systolic blood pressure, SBP; diastolic blood pressure, DBP; right and left ankle-brachial index, ABI).

EAT was easily visualized and clearly distinguishable from pericardial fat tissue (Figure 2A,B) [17]. EAT thickness strongly correlated with weight (*r* = 0.74, *p* < 0.001) and BMI (*r* = 0.79, *p* < 0.001), and moderately correlated with BSA (*r* = 0.69, *p* < 0.001), total fat (in Kg and in %; *r* = 0.61 and *r* = 0.491, respectively, *p* < 0.001), waist circumference (*r* = 0.65, *p* < 0.001), hip circumference (*r* = 0.53, *p* < 0.001), and WHR (*r* = 0.38, *p* < 0.001) (Figure 3). With regard to blood pressure and circulating cardiovascular risk factors, EAT thickness was moderately correlated with systolic pressure (*r* = 0.32, *p* < 0.001), triglycerides (*r* = 0.44, *p* < 0.001), HbA1c (*r* = 0.35, *p* < 0.001), and HDL cholesterol (*r* = −0.39, *p* < 0.001), and weakly correlated with diastolic pressure (*r* = 0.27, *p* = 0.006) and LDL cholesterol (*r* = 0.20, *p* = 0.041). Finally, EAT thickness was moderately correlated with IMT (*r* = 0.52, *p* < 0.001) and right ABI (*r* = 0.31, *p* = 0.002), and weakly correlated with left ABI (*r* = 0.28, *p* = 0.027) (Figure 3).

After the adjustment for age and gender, the multiple linear regression model, including cardiovascular risk factors found to be correlated with EAT thickness in bivariate analysis (BMI, total fat, BSA, WHR, LDL cholesterol, HDL cholesterol, triglycerides, glycated hemoglobin, systolic and diastolic blood pressures), showed that only BMI remained significantly and positively associated with EAT thickness (β = 0.182; SE = 0.082; *p* = 0.030) (Table 1).

### 3.5. Associations of Anthropometric Measures and Cardiovascular Parameters with Intima-Media Thickness (IMT)

IMT moderately correlated with weight (*r* = 0.42, *p* < 0.001), BMI (*r* = 0.46, *p* < 0.001), BSA (*r* = 0.38, *p* < 0.001), total fat (in kg and in %; *r* = 0.38 and *r* = 0.36, respectively, *p* < 0.001), waist circumference (*r* = 0.45, *p* < 0.001), hip circumference (*r* = 0.35, *p* < 0.001), and WHR (*r* = 0.31, *p* < 0.001). With regard to circulating cardiovascular risk factors, IMT was moderately correlated with triglycerides (*r* = 0.32, *p* < 0.001), HbA1c (*r* = 0.34, *p* < 0.001), and HDL cholesterol (*r* = −0.36, *p* < 0.001), and weakly correlated with LDL cholesterol (*r* = 0.22, *p* = 0.035) (Figure 3). However, the age- and gender-adjusted linear regression model, including cardiovascular risk factors found to be correlated with IMT in bivariate analysis (BMI, total fat, BSA, WHR, LDL cholesterol, HDL cholesterol, triglycerides, and glycated hemoglobin), showed that none of the above factors remained significantly associated with EAT thickness (Table 1).

### 3.6. The Association of Ideal Cardiovascular Health with EAT Thickness and IMT

**Figure 4:**
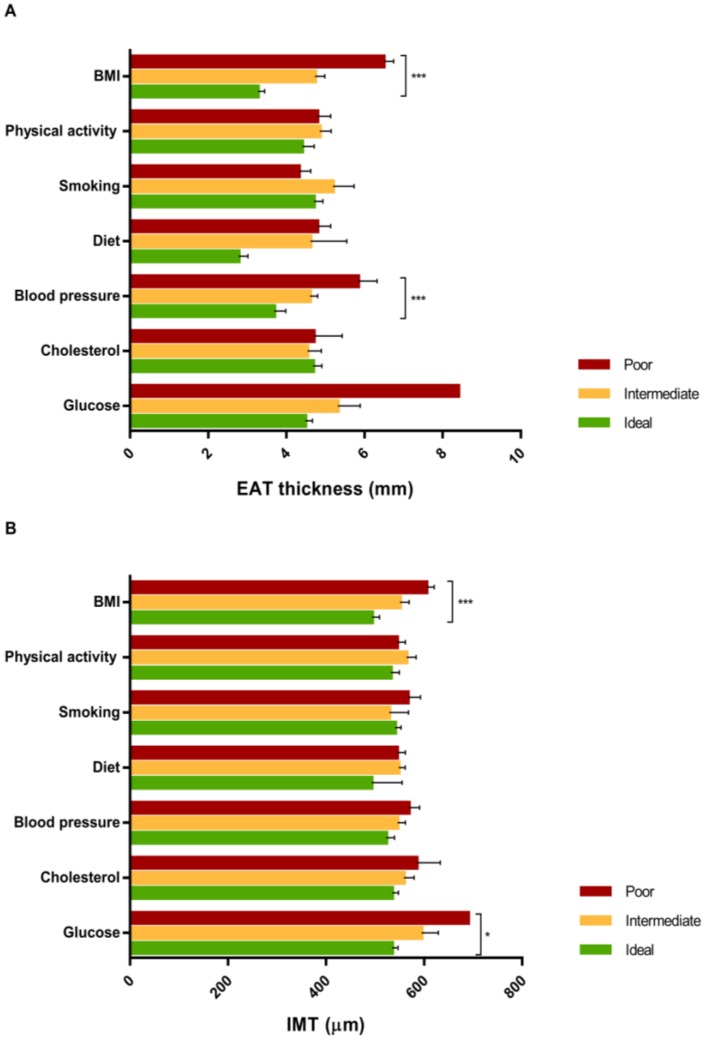
Age-adjusted means of EAT thickness (**A**) and IMT (**B**) according to ideal Cardiovascular Health (CVH) metrics.

EAT thickness was negatively correlated with CVH score (*r* = −0.45, *p* < 0.001) and its values, according to categories of each of the seven CVH components, were reported in Figure 4A; particularly, EAT thickness significantly decreased from the worst to the best category of BMI and blood pressure components (*p*-values < 0.001). In the unadjusted linear regression model, for every 1-unit increase in overall CVH score (indicating better cardiovascular health category), EAT thickness decreased by 0.317 mm (SE = 0.072; *p* < 0.001). This association was slightly reduced after adjusting for age and gender (β = −0.262; SE = 0.077; *p* = 0.001). Interestingly, when the seven CVH metrics were separately included in the regression model, only BMI and blood pressure components were significantly associated with EAT thickness in both the unadjusted and age- and gender- adjusted models (for BMI β_unad_j = −1.359; SE = 0.185; *p* < 0.001 and β_adj_ = −1.305; SE = 0.194; *p* < 0.001; for blood pressure β_unadj_ = −0.608; SE = 0.199; *p* = 0.003; β_adj_ = −0.607; SE = 0.206; *p* = 0.004, respectively).

Similarly, IMT was negatively correlated with CVH score (*r* = −0.38, *p* < 0.001) and its values, according to categories of each of the seven CVH components, are reported in Figure 4B; particularly, IMT significantly decreased from the worst to the best category of BMI and glucose components (*p* < 0.001 and *p* = 0.045, respectively). In the unadjusted linear regression model, for every one-unit increase in overall CVH score (indicating better cardiovascular health category), IMT decreased by 15.881 µm (SE = 5.002; *p* = 0.002). In addition, when the seven CVH components were separately included in the regression model, only the BMI component (β_unadj_ = −46.657; SE = 16.166; *p* = 0.005) was significantly associated with IMT. However, after adjustment for age and gender, neither overall CVH score nor CVH components were significantly associated with IMT (*p*-values > 0.05).

## 4. Discussion

EAT is an easily-measurable visceral adipose tissue that has physiological functions and pathological effects: in the healthy state, EAT releases cardioprotective cytokines and chemokines to the coronary vasculature, and may act as a fatty acid sink and energy store for the heart; in disease states, EAT releases inflammatory cytokines, and has been linked to multiple cardiometabolic conditions [6]. On the other hand, carotid IMT as measured by vascular ultrasound imaging has been shown to predict CVD risk in multiple large studies and meta-analysis [20]. IMT is an important atherosclerotic risk marker, but its increase can also be the result of nonatherosclerotic processes such as smooth muscle cell hyperplasia and fibrocellular hypertrophy, leading to medial hypertrophy and compensatory arterial remodeling [21]. Here, we examined the relationship between ideal CVH, EAT thickness, and IMT in a representative subset of individuals from the Kardiovize Brno 2030 cohort [13].

Given the disease-association of IMT and EAT, the first main objective of the present study was to determine the possible impact of age and sex on IMT and EAT thickness and their relationship with anthropometric measures and CVH parameters. We found increased IMT and EAT thickness with age, consistent with previous studies [22,23], but did not observe a sex-specific difference in these phenomena. A study on the Framingham Heart cohort suggested that EAT thickness was more associated with cardiovascular risk factors in women than in men [24]; however, other studies on the same cohort did not confirm this association [25]. Conversely, the Cardiovascular Risk in Young Finns Study, conducted in 2265 subjects aged 24–39 years, showed higher IMT values in men than in women [22]. Although age-related changes in sexual hormones might explain the detrimental effect of EAT on cardiac function in elderly women, at present, it remains unclear whether age-related and gender-related changes impact IMT and EAT thickness.

A growing body of evidence indicates that EAT thickness is also independently associated with traditional cardiovascular parameters, such as blood pressure and LDL cholesterol [18]. Our data confirmed the well-known impact of increased EAT thickness on blood lipid profiles, characterized by higher levels of triglycerides and lower levels of HDL cholesterol. We also showed a weak but significant positive correlation with both systolic and diastolic blood pressure, which confirms the association between EAT thickness and arterial hypertension [23,26]. Moreover, in our study, EAT thickness positively correlated with serum levels of glycated hemoglobin (HbA1c), a well-known diagnostic marker of type II diabetes. Consistently, increased EAT thickness was associated with impaired fasting glucose and diabetes mellitus [27].

The relationship between EAT and atherosclerosis is under debate [28]. IMT, measured by ultrasound, is instead a well-established indicator of subclinical atherosclerosis and is used as a novel marker of cardiovascular risk [29]. The first evidence of a possible link between EAT and IMT was demonstrated by Iacobellis et al. in human immunodeficiency virus–infected patients [30]. To our knowledge, this study is the first to demonstrate a moderate correlation between EAT thickness and IMT, in a population with no previous history of CVD. This is particularly significant because it points to EAT thickness as an additional predictor of subclinical atherosclerosis.

Obesity is surely among the well-established causes of ectopic fat deposition, and several studies demonstrated the relationship between BMI and EAT thickness [31,32]. We found that EAT thickness significantly correlated with anthropometric features; in particular, the strongest correlations were with waist circumference, WHR, and BMI. These relations are in agreement with findings summarized in recent systematic reviews and meta-analyses [33,34].

Interestingly, in a multiple linear regression model, only BMI showed an independent association with increased EAT thickness, but not with IMT. The finding on EAT partially confirms previous studies which demonstrated that EAT thickness was related to BMI and regional fat distribution [16]. Although EAT has been proposed as an independent predictor of visceral adiposity [16,33,35], results from the Framingham Heart Study Offspring cohort showed that EAT, but not visceral fat area, was associated with cardiovascular events after adjusting for conventional measures of obesity [25]. In this context, it is not possible to exclude differences related to the distribution of body fat depots that may characterize our Central European population-based cohort compared to other ethnic groups. The power of EAT versus visceral area and/or BMI as independent predictors of CVD is unclear and it remains under scrutiny. On the other hand, the observed lack of association between IMT and BMI is consistent with previous findings [36].

While increased IMT was shown as a significant outcome in individuals with poor CVH by Kulshreshtha et al. [37], prior to this study, it has not been proven that poor CVH could affect EAT thickness. The present study demonstrated that overall CVH score, as well as two CVH metrics (i.e., BMI and blood pressure), are associated with EAT thickness and IMT. However, after adjustment for age and gender, the association was confirmed for EAT thickness but not for IMT. Since the study by Kulshreshtha et al. was conducted on 490 male twins [37], further research should evaluate the effect of age and sex on this relationship. Overall, our findings suggest that EAT thickness, but not IMT, should be considered as an independent echocardiographic outcome of poor CVH.

The main limitations of our study are the limited sample size and the cross-sectional nature of the study that does not allow us to demonstrate the causality of the relationship; however, it seems reasonable that changes in EAT thickness and IMT are a consequence and not a cause of poor CVH. Even though the sub-sample was not representative of the Kardiovize Brno cohort in terms of age and gender, the age- and sex-adjusted relationships might be generalized to the entire Central European population. However, the cross-sectional nature of our analyses does not allow us to yet understand the mechanisms that lead to increased IMT and EAT thickness in subjects with poor CVH. Moreover, all data were based on a single measurement and may not reflect changes over time. Additional and larger prospective studies on IMT and EAT thickness and their relationships with cardiovascular risk factors are warranted.

## 5. Conclusions

We demonstrated for the first time that CVH is associated with EAT thickness. Interestingly, this relationship seems to be dependent on BMI and blood pressure rather than on the other CVH metrics. However, further research is encouraged to confirm whether monitoring CVH could be a suitable strategy to stratify subjects according to their EAT thickness, targeting these individuals for CVH promotion and disease reduction.

## Figures and Tables

**Table 1:** Linear regression analyses of epicardial adipose tissue thickness and intima-media thickness with anthropometric measures and cardiovascular parameters, adjusted for age and gender.

Linear Regression Model	EAT Thickness		IMT	
	B (SE)	*p*-Value *	B (SE)	*p*-Value *
BMI	0.182 (0.082)	0.030	0.081 (0.095)	0.128
Total fat	0.007 (0.027)	0.787	0.005 (0.027)	0.678
BSA	0.184 (1.234)	0.882	0.126 (1.229)	0.691
WHR	0.897 (2.154)	0.678	0.876 (2.100)	0.531
LDL cholesterol	0.022 (0.178)	0.901	0.008 (0.177)	0.987
HDL cholesterol	−0.144 (0.397)	0.719	−0.110 (0.394)	0.697
Triglycerides	−0.051 (0.264)	0.849	−0.020 (0.257)	0.902
Glycated hemoglobin	−0.024 (0.034)	0.480	−0.023 (0.033)	0.415
Systolic blood pressure	0.031 (0.025)	0.221	-	-
Diastolic blood pressure	−0.028 (0.034)	0.411	-	-

* *p*-values < 0.05 are in bold font. Abbreviations: EAT, epicardial adipose tissue; IMT, Intima-media thickness; BMI, Body mass index; BSA, Body surface area; WHR, Waist-Hip ratio; HDL, high density lipoprotein; LDL, low density lipoproteins.

## Supplementary Materials

The following are available online at http://www.mdpi.com/2077-0383/7/5/113/s1. Supplemental Table S1. Anthropometric measures and cardiovascular parameters of the study population by gender.
