# Supplementary material for: Association of Cardiovascular Health with Epicardial Adipose Tissue and Intima Media Thickness: The Kardiovize Study
Source: J Clin Med. 2018 May 13;7(5):113. doi: 10.3390/jcm7050113 (PMC5977152; doi:10.3390/jcm7050113)
Supplement: Supplementary file 1 [file jcm-07-00113-s001.pdf]

Supplemental Table 1. Anthropometric measures and cardiovascular parameters of the study population by gender

Characteristics	Men		Women		p-value*
	Mean	SEM	Mean	SEM	
**Age (years)**	38.51	1.02	44.41	2.02	**0.004**
**Height (cm)**	182.24	0.75	170.79	1.01	**<0.001**
**Weight (Kg)**	89.78	1.96	76.52	2.62	**<0.001**
**BMI (Kg/m^2)**	27.42	0.60	26.25	0.80	0.251
**BSA (m^2)**	2.16	0.03	1.90	0.04	**<0.001**
**Total fat (Kg)**	19.95	1.37	24.31	1.84	0.064
**Waist (cm)**	96.65	1.59	84.68	2.12	**<0.001**
**Hip (cm)**	105.13	1.17	107.18	1.57	0.305
**WHR**	0.92	0.01	0.79	0.01	**<0.001**
**Systolic blood pressure (mmHg)**	125.70	1.11	122.92	1.48	0.144
**Diastolic blood pressure (mmHg)**	75.72	0.80	75.17	1.08	0.662
**Glucose (nmol/l)**	5.03	0.07	4.77	0.09	**0.030**
**Total Cholesterol (nmol/l)**	4.87	0.12	4.79	0.17	0.681
**LDL cholesterol (nmol/l)**	3.02	0.10	2.80	0.13	0.203
**HDL cholesterol (nmol/l)**	1.31	0.05	1.72	0.07	**<0.001**
**Triglycerides (nmol/l)**	1.33	0.08	0.99	0.11	**0.012**
**Glycated hemoglobin (nmol/mol)**	38.84	0.59	38.31	0.79	0.597
**Creatinine (nmol/l)**	13.37	0.74	10.23	0.99	**0.015**
**QIMT (µm)**	559.98	11.38	519.39	15.97	**0.046**
**Right ABI**	1.03	0.01	1.03	0.02	0.691
**Left ABI**	1.03	0.01	1.03	0.02	0.807

*p-values<0.05 are in bold font

Abbreviations: SEM, standard error of the mean; BMI, Body mass index; BSA, Body surface area; WHR, Waist-Hip ratio; HDL, high density lipoprotein; LDL, low density lipoprotein; QIMT, Quality Intima-media thickness; Ankle-Brachial Index
